# Quantifying model evidence for yellow fever transmission routes in Africa

**DOI:** 10.1371/journal.pcbi.1007355

**Published:** 2019-09-23

**Authors:** Katy A. M. Gaythorpe, Kévin Jean, Laurence Cibrelus, Tini Garske

**Affiliations:** 1 School of Public Health, Imperial College London, London, United Kingdom; 2 Laboratoire MESuRS, Conservatoire National des Arts et Métiers, Paris, France; 3 Infectious Hazard Management Department, World Health Organisation, Geneva, Switzerland; 4 Unité PACRI, Institut Pasteur, Conservatoire National des Arts et Métiers, Paris, France; 5 MRC Centre for Global Infectious Disease Analysis, Department of Infectious Disease Epidemiology, Imperial College, St Mary’s Campus, London, United Kingdom; The Pennsylvania State University, UNITED STATES

## Abstract

Yellow fever is a vector-borne disease endemic in tropical regions of Africa, where 90% of the global burden occurs, and Latin America. It is notoriously under-reported with uncertainty arising from a complex transmission cycle including a sylvatic reservoir and non-specific symptom set. Resulting estimates of burden, particularly in Africa, are highly uncertain. We examine two established models of yellow fever transmission within a Bayesian model averaging framework in order to assess the relative evidence for each model’s assumptions and to highlight possible data gaps. Our models assume contrasting scenarios of the yellow fever transmission cycle in Africa. The first takes the force of infection in each province to be static across the observation period; this is synonymous with a constant infection pressure from the sylvatic reservoir. The second model assumes the majority of transmission results from the urban cycle; in this case, the force of infection is dynamic and defined through a fixed value of *R*_0_ in each province. Both models are coupled to a generalised linear model of yellow fever occurrence which uses environmental covariates to allow us to estimate transmission intensity in areas where data is sparse. We compare these contrasting descriptions of transmission through a Bayesian framework and trans-dimensional Markov chain Monte Carlo sampling in order to assess each model’s evidence given the range of uncertainty in parameter values. The resulting estimates allow us to produce Bayesian model averaged predictions of yellow fever burden across the African endemic region. We find strong support for the static force of infection model which suggests a higher proportion of yellow fever transmission occurs as a result of infection from an external source such as the sylvatic reservoir. However, the model comparison highlights key data gaps in serological surveys across the African endemic region. As such, conclusions concerning the most prevalent transmission routes for yellow fever will be limited by the sparsity of data which is particularly evident in the areas with highest predicted transmission intensity. Our model and estimation approach provides a robust framework for model comparison and predicting yellow fever burden in Africa. However, key data gaps increase uncertainty surrounding estimates of model parameters and evidence. As more mathematical models are developed to address new research questions, it is increasingly important to compare them with established modelling approaches to highlight uncertainty in structures and data.

## Author summary

Yellow fever (YF) is notoriously under reported due to non-specific symptom spectrum and the true burden is highly uncertain as a result of a complex transmission cycle. As such, estimates surrounding YF burden are highly uncertain and the mechanisms behind transmission are often unclear. We assess these mechanisms and the resulting uncertainty by estimating two existing models of YF transmission within a product space framework. This allows us to produce updated estimates of transmission intensity and to compare the relative support for each model given the data. We find strong support for a model assuming a static force of infection, approximating the constant infection pressure from the sylvatic reservoir of YF. We also highlight areas where data is sparse, often the same areas estimated to have especially high transmission intensity. This is the first robust multi-model approach to applied YF modelling and provides a framework that could be extended to other disease models.

## Introduction

Yellow fever (YF) is a vector-borne viral disease estimated to cause 78,000 deaths in Africa alone [1]. It is endemic to both Latin America and Africa although as much as 90% of cases are thought to occur in the latter. The disease has a wide spectrum of severity ranging from asymptomatic infection to severe disease including jaundice and haemorrhaging [2]. Particularly in milder cases, the disease is fairly non-specific and many individuals may be mistaken for having other highly prevalent infections such as influenza or malaria.

The main urban vector of yellow fever transmission is *Aedes aegypti*, also a carrier of Dengue and Zika [3]. However, there are a number of intermediate and sylvatic vectors which link the three main transmission cycles involving humans and non-human primates. The two main transmission cycles are sylvatic, where infection occurs mainly in non-human primates and is driven by tree-hole-breeding mosquitoes such as *Aedes africanus*, and urban, where transmission is mainly in humans and is driven by *Aedes aegypti* [3, 4]. A third cycle cycle can exist in the moist Savannah region of Africa when the tree-hole-breeding mosquito densities reach high levels, infecting both humans and non-human primates. The various cycles have different dynamics. When infection reaches urban settings, explosive outbreaks can occur due to the high density of human hosts and mosquitoes. Yet, the consistent infection pressure from the sylvatic reservoir of disease leads to speculation that many yellow fever cases arise as a result of this cycle. Although, this pressure may vary temporally due to environmental factors. The third cycle, an intermediate form of transmission, affects humans living or working in the jungle border areas and may exhibit dynamics of both extremes. This naturally leads to questions concerning the optimal routes for controlling the disease.

Eradication of yellow fever is not feasible due to the sylvatic reservoir. However, there exists a human vaccine which was developed in the 1930s and is estimated to have effective protection that lasts a lifetime [5]. As such, control of the disease is possible and recent mass vaccination campaigns have been found to reduce annual burden by 57% across the targeted countries [1]. Yet assessing the impact of such an intervention will always be troubled by uncertainty in the prevalence and dynamics of the disease.

A great deal of uncertainty is inherent in yellow fever modelling. The varying level of symptoms experienced by infected individuals lead to significant under or mis-reporting. This is further complicated by the surveillance structures and remoteness of some of the communities at risk from yellow fever. Furthermore, there is little data concerning the non-human primates who act as the environmental reservoir of the disease. The relationship between different primate species, mosquito types and the human hosts can vary the severity of transmission. Tackling the uncertainty in model structures as well as parameters gives a start point from which to begin quantifying the importance of transmission routes for this virus.

The uncertainty in yellow fever understanding leads to a number of challenges. In Africa, little is known about YF in non-human primates (NHP); because of this, it is difficult to assess how dynamics in NHP may affect epizootic events. Furthermore, the relative contribution of different transmission cycles to the burden of YF in humans is unclear from the limited data available. As a result, specific transmission-targeted intervention, which could allow for effective and cost-efficient control of the disease, is difficult to justify.

There have been several approaches to modelling YF, often without explicit assumptions concerning the transmission cycles involved. Locally, Monath and Nisidi developed models to assess the benefits of YF vaccination in Nigeria, finding it to be a cost-effective addition to the enhanced programme of immunization (EPI) [6]. They assumed that infection in humans occurred purely as a result of contact with the sylvatic reservoir; however they suggested a background prevalence of immunity of 60% may preclude epidemic spread. Kraemer et al. modelled the geographic spread of the YF outbreak in Angola and Democratic Republic of Congo 2016 using human mobility data and a measure of vector suitability in order to prioritise districts for intervention. On a global scale, Shearer et al. modelled the vaccination coverage and then YF risk by incorporating distribution maps of reservoir and vector species as well as habitat suitability [7, 8]. We focus on two models for transmission in Africa. The first is that of Garske et al. which is used to inform and assess vaccination strategy from GAVI and the Gates foundation [1]. This model assumes the force of infection is static resulting from a constant infection pressure from the sylvatic reservoir and uses a generalised linear model of YF occurrence to extrapolate transmission intensity to areas where data is sparse. Our second focus model is that of Jean et al. which extends the work of Garske et al. to account for a form of herd immunity [9]. In this case, infection is assumed to be human-human mediated by mosquitos thus representing only the urban transmission cycle. Both represent an extreme version of yellow fever transmission; however, within our averaging framework, we will assess the relative evidence for each model formulation and thus compare the role of each transmission cycle. This approach differs from Faria et al. who used genomic data as well as the age/ sex distributions of infected cases to find that a YF outbreak in Minas Gerais was mainly due to sylvatic transmission [10].

In this manuscript we shall detail the updated data sources and describe in brief the two highlighted model structures for yellow fever in Africa [1, 9]. We shall then define a composite model incorporating both model formulations and estimate it within a Bayesian framework to evaluate key values of model selection such as Bayes factors. This will allow us to produce predictions incorporating both model approaches and the inherent uncertainty of multiple model structures and parameters. This is the first rigorous multi-model approach applied to yellow fever.

## Materials and methods

### Overview

**Fig 1:**
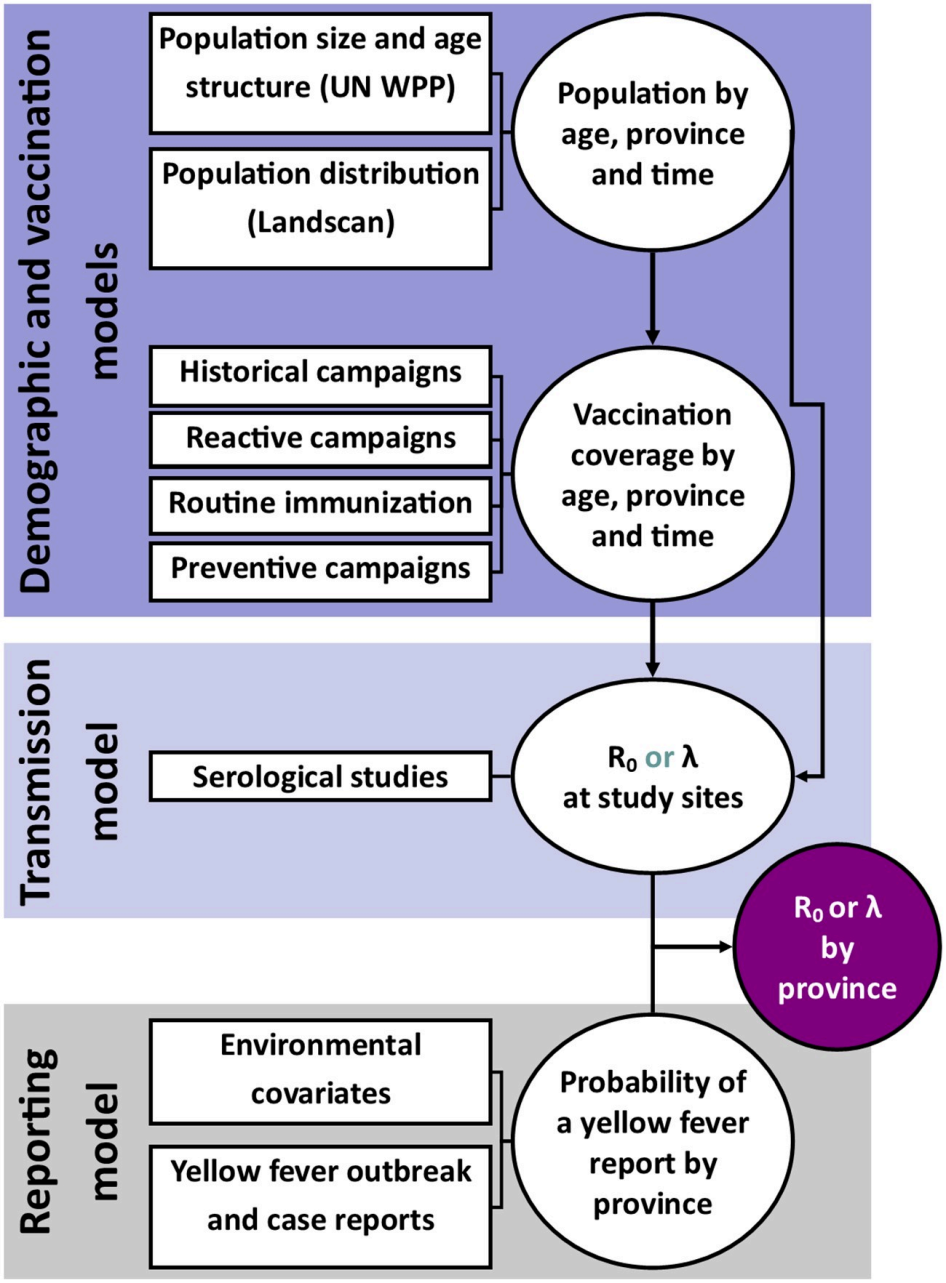
Diagram of models and data sources where *R*_0_ denotes the basic reproduction number and λ, the force of infection. Circles denote a product of calculation or inference; square boxes denote data sources.

As described in [1], we estimate a generalised linear model at locations where yellow fever was reported from 1987 to 2017. This model predicts, for each location, the probability of at least one yellow fever report over the observation period based on covariates such as the temperature or land cover type. Then, given a probability of detection for each country informed by the mismatch between case reports and serology, the number of cases that would give rise to this probability of report is estimated. Finally, this number of infections is converted to either a force of infection, λ, see [1], or a basic reproduction number estimate, *R*_0_, see [9]. Fig 1 shows the various data sources for both models.

### Data

The models were estimated at the resolution of the first administrative unit, often called a province. All data detailed below is resolved or aggregated at that level unless stated otherwise. This resolution was optimal for the YF occurrence data, other inputs such as environmental covariates were originally gridded.

#### Yellow fever occurrence

A database of the provinces where outbreaks of yellow fever have been reported was compiled from 1984 to 2017. This was informed by data from the Weekly epidemiological record (WER), the WHO disease outbreak news (DON), the published literature and internal WHO outbreak records.

Case-based surveillance for YF was established in 21 West and Central African countries and is recorded in the yellow fever surveillance database (YFSD) held by the African regional office of the World Health Organisation (AFRO). This database is designed to use a very broad case definition (jaundice or haemorrhage with fever) to ensure high sensitivity (at the cost of low specificity of suspected cases). That means that most reported cases are not due to YF and only 1-2% will be lab-confirmed as YF. In addition to using the confirmed cases to inform the occurrence of YF, we use the above data as a proxy for surveillance effort in each country. Assuming an approximately constant incidence of the syndrome ‘fever with jaundice and/or haemorrhage’, the per-capita incidence of reported suspected cases yields information on the surveillance effort. We therefore aggregate this measure at country level and divide by the national population to be used as a covariate in the generalised linear model.

#### Vaccination coverage

We use the methodology of Garske et al. with updated data sets to estimate vaccination coverage [1]. This methodology is also described and visualised in finer detail in the Polici shiny application where vaccine coverage is visualised at province level from 1940 to 2050 for the 34 endemic countries in Africa. [11]. This data may also be downloaded for each country. The coverage is based historic data on large-scale mass vaccination activities [12, 13], outbreak response campaigns as reported in the WHO weekly epidemiological record and disease outbreak news [14, 15], recent preventive mass-vaccination campaigns conducted as part of the yellow fever initiative [16] and routine infant vaccination reported by the WHO/UNICEF estimates of national immunization coverage (WUENIC) [17].

#### Serological surveys

**Fig 2:**
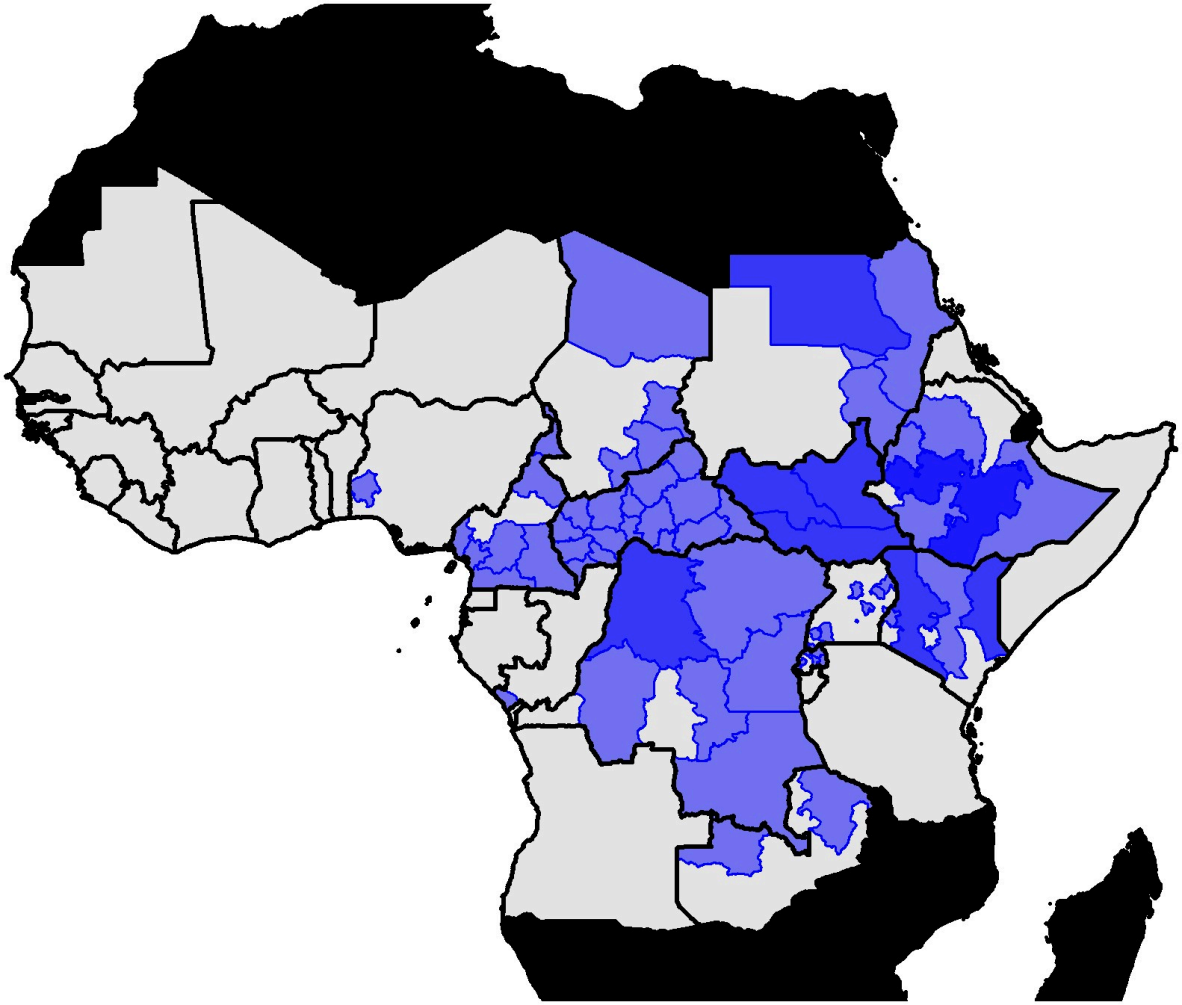
Location of serological surveys used in the models. The colour intensity indicates number of studies covering each province where grey = 0, pale blue = 1 and darker blue = 2 or more. Further details on serological surveys are available in Table A in S1 Text [1, 9, 18–23]. Maps were produced from GADM version 2.0.

We use serological surveys to assess transmission intensity in specific regions of the African endemic zone, the locations are displayed in Fig 2. Theses include published and unpublished surveys [18–23]. The unpublished surveys were from various East African countries performed between 2012 and 2015 as part of the YF risk assessment process [24]. Of the published surveys, included in the estimation of Garske et al. and Jean et al. we omit that of the Central African Republic as it may capture post-outbreak dynamics and thus not represent the population serological status at steady state [9, 18, 25]. In the majority of surveys, individuals who were known to have been vaccinated were excluded; however in south Cameroon the vaccination status was unknown so we estimate an additional vaccination factor [19]. This factor represents the probability that a vaccinated individual is included in the study. Further summary characteristics of the surveys are included in the Table A in S1 Text.

#### Demographic data

For each country, the population size and age structure was obtained from the UN World Population Prospects (UN WPP) [26]. These were then disaggregated from the five- year age band into annual birth cohorts according to the method described in [25]. We combine the estimates with spatial population distributions from LandScan 2014 [27] in order to estimate the populations sizes at province level assuming the same age structure across all provinces in a given country. Landscan provides population size estimates for approximately 1 km square pixels. Combining these data sets we arrive at the total number of individuals in each age group and province over time assuming that spatial distributions are fairly static.

#### Environmental data

The generalised linear model includes environmental covariates to account for the dependence of transmission on factors such as temperature and land cover. These data sets specifically include the enhanced vegetation index (EVI), middle infra-red reflectance (MIR), land cover types, rainfall estimates, temperature and altitudes [28–30]. These are gridded data at various resolutions, ranging from approximately 1km to 10km, which we aggregated to province level by calculating the mean value weighted by the population size at each grid cell.

### Models

#### Generalised linear model of yellow fever reports

This approach is common to both models and described in full in [1]. The generalised linear model is estimated from occurrence data which we assume to be binomially distributed. The model predictions, *q*_*i*_ for each province or first administrative unit, *i*, are given by

$$\begin{array}{c} q=1-\text{exp}(-\text{exp}(X\beta )) \end{array}$$ (1)

where *X* = *X*_*ij*_ is the matrix of covariates used in the model, *i* denotes province and *j* denotes covariate, and *β* is the vector of parameters to be estimated. Garske et al. used a complementary log-log link function in order to have a more realistic interpretation in terms of surveillance quality when relating the GLM to transmission intensity, detailed later. The full list of covariates assess is detailed in [1]; however, in this paper we examine only the best-fitting model which incorporates aspects such as land cover type, temperature, surveillance quality, population size, enhanced vegetations index and longitude. This is the same for both formulations of the transmission model.

#### Relating occurrence to transmission intensity

In both of the model formulations, the transmission intensity produces an estimate of the number of infections in any year. As such we may calculate the number of infections over the reporting period. If we can also calculate the probability of report, the number of infections will link transmission intensity and the occurrence of yellow fever reports. First, we relate *q*_*i*_, the probability of at least one yellow fever report in province *i* over the observation time to *n*_*inf*,*i*_, the number of infections in that province, through a Poisson process for the detection on infection,

$$\begin{array}{c} q_{i}=1-{\left(1-\rho_{i}\right)}^{n_{\text{inf},\text{i}}} \end{array}$$

where *ρ*_*i*_ = *ρ*_*c*_ is the **per country** probability of detection which may be related to the GLM in following way

$$\begin{array}{c} n_{\text{inf},\text{i}}\;\text{ln}\left(1-\rho_{c}\right)=-\text{exp}\left(X\beta \right). \end{array}$$

Through the repeated taking of logarithms, the probability of detection may be written in terms of the GLM coefficients and a baseline surveillance quality, *b*,

$$\begin{array}{c} \text{ln}\left(n_{\text{inf},\text{i}}\right)=X\beta -\beta_{c}-b\;\text{and}\;\text{ln}\left(-\text{ln}\left(1-\rho_{c}\right)\right)=\beta_{c}+b \end{array}$$ (2)

which is calculated separately using serological survey data from the left part of Eq 2.

#### Transmission intensity as a static force of infection

In the areas where serological survey data is available, it is possible to estimate the transmission intensity assuming certain information about vaccination coverage and demography. In the first instance, Garske et al. assumed transmission occurred as a static force of infection, hereby termed the λ model [1]. This formulation may be likened to the assumption that most Yellow Fever infection pressure comes from the sylvatic reservoir and there are limited instances where infection reaches urban environments to be sustained in the human and vector populations alone.

The force of infection is assumed to be the same for all age groups within a province over time, so we may model the serological status of the population in age group *u* with force of infection λ as the following,

$$\begin{array}{c} S_{F}\left(\lambda ,u\right)=1-(1-\frac{\sum_{a\in u}(1-\text{exp}(-\lambda a))p_{a}}{\sum_{a\in u}p_{a}})(1-\frac{\sum_{a\in u}v_{a}p_{a}}{\sum_{a\in u}p_{a}}) \end{array}$$ (3)

where *p*_*a*_ denotes the proportion of the population of age *a* and *v*_*a*_ denotes the vaccination coverage at age *a*.

#### Transmission intensity related to the basic reproduction number

Jean et al. developed a formulation for the transmission dynamics of yellow fever that is based on the basic reproduction number [9]. Therefore, the model is dynamic and the force of infection may change depending on population immunity. In contrast to the the λ model, the basic reproduction number or *R*_0_ model assumes all infections are human-to-human, mediated through the main urban vector of Yellow Fever, *Aedes aegypti*. The ramification of this assumption is that the herd affects of any intervention measures will be captured by the *R*_0_ model and not by the λ model.

The *R*_0_ model assumes that each province is at endemic equilibrium. Each year the endemic equilibrium is maintained by the addition of new infections such that the fraction of susceptible population is equal to 1/*R*_0_. Thus,

$$\begin{array}{c} N_{inf}\left(t\right)=\text{max}\;(0,P_{tot}\left(t\right)(S\left(t\right)-\frac{1}{R_{0}})), \end{array}$$

where *N*_*inf*_(*t*) is the number of infections in year *t* and *P*_*tot*_(*t*) is the total population size in year *t*.

The immunity granted through vaccination is also incorporated into the model, reducing the number of infections occurring in any one year. However, seropositivity due to vaccination is included in only some of the serological studies. In this case, the natural and vaccinated serological status must be tracked separately and the resulting seroprevalence at age *a* in year *t* may be modelled as

$$\begin{array}{c} S_{R_{0}}\left(a,t\right)=1-i^{-}v^{-}\left(a,t\right) \end{array}$$ (4)

where *i*^−^ *v*^−^(*a*, *t*) denotes those individuals who are both uninfected and unvaccinated.

### Model estimation

We wish to average the model predictions of disease burden whilst taking into account the uncertainty in parameter estimates and model structure. As such, we would like to calculate the probability of each model given the data and uncertain parameter estimates. We do this by estimating the model evidence using Markov Chain Monte Carlo (MCMC) on the product or composite space of the models using the method of Carlin and Chibb [31–35]. They wished to develop a rigorous model selection, without affecting convergence, in the situation where the dimension of the parameter space is not fixed [31]. This method not only gives us an estimate of the model evidence but also allows us to calculate the Bayes factors and marginal posterior distributions for each parameter.

#### Method in brief

**Fig 3:**
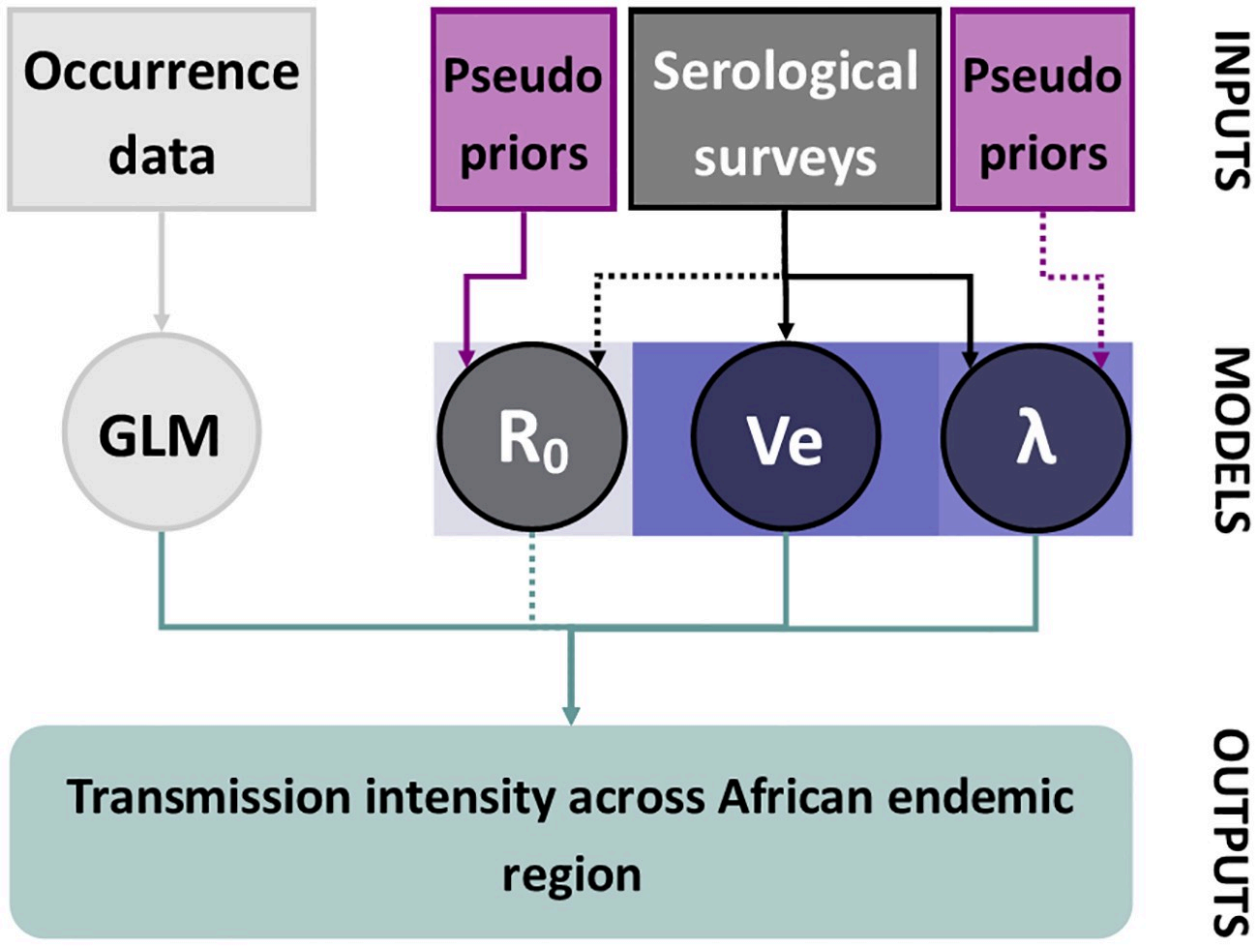
Diagram of model inference at one iteration. Aspects relating the λ model are contained in the blue box. There are two main data sets that the models are estimated from, occurrence data, shown in white, and serological data, shown in black. Elements contained within circles denote families of model parameters with the vaccine efficacy (Ve) parameter common to both transmission model formulations. When a model is not activated (*R*_0_ in this figure) the model-specific parameters are informed by pseudopriors, shown in purple. Solid arrows imply that the parameter is being informed by that data source or pseudoprior in this iteration; dashed arrows imply that the parameter family is currently not informed by that data source or pseudoprior in this iteration.

We examine the full space of parameters for all models, $\theta =(\theta_{R_{0}},\theta_{\lambda })$. Therefore, we define a composite model containing all parameters and a model indicator, *M*. The model indicator dictates which parameters are informed by the data at each iteration; when the model indicator ‘selects’ model A, we term the parameters corresponding to model A as **activated**. We sample the posterior of this composite model by proposing values of *θ* and *M*, and accepting them with a probability proportional to the prior distributions of *M* and activated model parameters; the likelihood, and pseudopriors. Pseudopriors can be considered as linking densities which are chosen to improve mixing between models and do not contribute to the marginal likelihoods. They act as place holders for the parameters that are not currently informed by the data and therefore only apply to inactivated parameters. In our models the model-specific parameters describe the transmission intensity in the serological surveys, namely λ and *R*_0_ estimates which may be activated or inactivated by *M*. The parameters that are common to both models, such as the vaccine efficacy, are always informed by the data and therefore always activated. These parameters do not have pseudopriors, see Fig 3 for links. The resulting posterior samples of model indicator *M* will give us an indication of which model is better supported by the data; the posterior samples of the shared parameters will indicate the full uncertainty in estimates given the two formulations.

#### Theoretical background

We may describe our *R*_0_ and λ models as Bayesian models defined by a joint probability distribution of data, *D*, and model parameters, *θ*:

$$\begin{array}{c} p\left(D,\theta_{i}|M_{i}\right)=p\left(D|\theta_{i},M_{i}\right)p\left(\theta_{i}|M_{i}\right). \end{array}$$

We define a mixture model of the two models where our parameter vector can take any value from the Cartesian product of the individual parameter spaces [32]. Therefore, the full model may be written as the following,

$$\begin{array}{c} p\left(D|\theta \right)=p\left(D,\theta |M_{\lambda }\right)p\left(M_{\lambda }\right)+p\left(D,\theta |M_{R_{0}}\right)p\left(M_{R_{0}}\right) \end{array}$$

where *p*(*M*_*i*_) is the prior for model *i* ∈ (λ, *R*_0_) and $p\left(M_{R_{0}}\right)+p\left(M_{\lambda }\right)=1$. Given the above formulation of the mixture model, the marginal likelihood for each model may be described with the following,

$$\begin{array}{c} p\left(D|M_{i}\right)=\int p\left(D|\theta_{i},M_{i}\right)p\left(\theta_{i}|M_{i}\right)d\theta_{i}. \end{array}$$

Note that the above is independent of the prior distribution *p*(*θ*_*j*_|*M*_*i*_), or pseudoprior, which means that *p*(*θ*_*j*_|*M*_*i*_) may be chosen specifically to improve mixing and that we may calculate the Bayes factors as the ratio of the two marginal likelihoods [31, 32].

We generate samples from the joint posterior distribution of the model parameters and indicator using Metropolis-within-Gibbs where the joint posterior is given by,

$$\begin{array}{c} p\left(M,\theta |D\right)\propto p\left(D|\theta_{i},M_{i}\right)p\left(\theta_{i}|M_{i}\right)p\left(\theta_{j}|M_{i}\right)p\left(M_{i}\right). \end{array}$$

Following Carlin and Chibb and, separately, Lodewyckx et al. [31, 32], we use a Gibbs sampler. However, in both of the above, the likelihood, *p*(*D*|*θ*_*i*_, *M*_*i*_), could be sampled easily, whereas we use a pseudo-likelihood assuming observations are independent, with form shown in Eq 6. As such, where the likelihood occurs in the Gibbs sampling algorithm, we include a Metropolis-Hastings step. This means that we propose new parameter values from a symmetric distribution which we then accept with a probability proportional to the likelihood and prior distributions at that point.

The steps for one iteration of this approach are as follows:

1. We sample new parameters given the current value of the model indicator from the conditional distributions given by
  $$\begin{array}{c} p\left(\theta_{j}|\theta_{i\neq j},M,D\right)\propto \{\begin{array}{cc} p\left(D|\theta_{j},M_{j}\right)p\left(\theta_{j}|M_{j}\right)\; & M=M_{j},\\ p(\theta_{j}|M_{i}) & M=M_{i\neq j}. \end{array} \end{array}$$
  **Inactivated parameters** are sampled directly from their pseudopriors, *p*(*θ*_*j*_|*M*_*i*_). The pseudopriors are set to proper distributions that have been fitted to the marginal posterior distributions of individual model runs.
  **Activated parameters** are informed by the likelihood and prior distributions and so are sampled using a Metropolis-Hastings step. As such, new values, denoted with a star, are proposed from a multivariate normal transition kernel and accepted with probability proportional to,
  $$\begin{array}{c} \text{min}\;(1,\frac{p\left(D|\theta_{j}^{*},M_{j}\right)p\left(\theta_{j}^{*}|M_{j}\right)}{p\left(D|\theta_{j},M_{j}\right)p\left(\theta_{j}|M_{j}\right)}). \end{array}$$
2. We sample the model indicator given the new values of the parameters from the conditional distributions denoted by
  $$\begin{array}{c} p\left(M_{i}|\theta ,D\right)\propto p\left(D|\theta_{i},M_{i}\right)p\left(\theta_{i}|M_{i}\right)p\left(\theta_{j}|M_{i}\right)p\left(M_{i}\right). \end{array}$$
  This is completed as a Metropolis-Hasting step with new model indicator value proposed from symmetric distribution (in our case, either model is chosen with equal probability) and accepted with a probability proportional to the ratio of the conditional distributions of the new and old model indicator.

Finally, we may approximate the posterior probability of each model as the proportion of iterations accepted for that model, ie.

$$\begin{array}{c} \hat{p}\left(M_{k}|D\right)=\frac{\text{Number}\;\text{of}\;\text{iterations}\;\text{spent}\;\text{on}\;M_{k}\;}{\text{Total}\;\text{number}\;\text{of}\;\text{iterations}}. \end{array}$$ (5)

#### Specific formulation

Our two model formulations retain the generalised linear model of yellow fever occurrence which is used to extrapolate transmission intensity across the entire African endemic region. To estimate this, we use binary occurrence data to produce the following likelihood,

$$\begin{array}{c} \text{ln}\;L_{GLM}=\sum_{i}(y_{i}\;\text{ln}\left(q_{i}\right)+\left(1-y_{i}\right)\;\text{ln}\left(1-q_{i}\right)), \end{array}$$

where *y*_*i*_ denote yellow fever reports in province *i* over the observation period and *q*_*i*_ is the predicted probability of a report given by Eq 1. We estimate the GLM with MCMC and a Metropolis-Hasting step based on an adaptive proposal distribution.

The difference between the λ and *R*_0_ model formulations occurs when predicting seroprevalence. However, the serological study likelihoods have the same general form. The positive samples are assumed to be binomially distributed so we have the following pseudo-log-likelihood,

$$\begin{array}{c} \text{ln}\;L_{SERO}=\sum_{u}\text{ln}\;(\left(\begin{array}{c} n_{\text{tot},\text{u}}\\ n_{\text{pos},\text{u}} \end{array}\right)\;S_{M}{\left(T,u\right)}^{n_{\text{pos},\text{u}}}{\left(1-S_{M}\left(T,u\right)\right)}^{n_{\text{tot},\text{u}}}), \end{array}$$ (6)

where *S*_*M*_(*T*, *u*) denotes the seroprevalence predicted by model *M* ∈ (λ, *R*_0_) for age group *u* and transmission type *T* ∈ (λ, *R*_0_) given by Eqs 3 and 4; *n*_tot,u_ and *n*_pos,u_ denote the total number of samples and positive samples in the data respectively.

As we have overlapping parameters which will be activated irrespective of the value of the model indicator, we only need to define pseudopriors for the parameters that differ between models i.e., *R*_0_ and λ. We choose the pseudopriors to be approximations of the individual model posterior predicted values from previous MCMC runs, see the appendix for all distributions. These are calculated using the package fidistr in R assuming the posteriors of individual model runs are normally distributed, see supplementary material for fits. We adjust the variance of these fitted distributions in order to ease mixing between the two models as the pseudopriors do not affect the marginal posterior distributions of the models. This was performed by adjusting a scaling factor for the standard deviation for the *R*_0_ pseudoprior to ensure that the values of the pseudopriors applied to the median values of the posterior outputs of both models have a difference close to zero.

Each model has a prior which may affect the mixing between models and the final posterior model probability. As such, we choose this to be such that each model is activated relatively frequently to ensure efficient sampling of the parameter space. We examined the posterior probabilities given either value of the model indicator and adjusted the model prior information such that the difference was minimal. In our case, and primarily due to the difference in model likelihood for the median posterior value of each parameter taken from initial individual model estimation runs, the model prior for the λ model is *π*_λ_ = exp(−10). The model prior for the *R*_0_ is $\pi_{R_{0}}=1-\pi_{\lambda }$. The final Bayes factors will take into account the prior model probabilities in the following way:

$$\begin{array}{c} B_{R_{0}\lambda }≃\frac{\hat{p}\left(M_{R_{0}}|D\right)\pi_{\lambda }}{\hat{p}\left(M_{\lambda }|D\right)\pi_{R_{0}}}. \end{array}$$ (7)

### Bayesian model averaging

Once we have estimated the model evidence and approximated the Bayes factors, we may produce average disease burden predictions given both of our models. We aim to predict the burden of yellow fever in the African endemic zone for the year 2018 which may be calculated using the posterior predicted distribution of the risk of a yellow fever report and either the posterior distributions for λ or *R*_0_ in the serological survey areas. We consider a pool of possible predictions from both model formulations to arrive at the average model prediction. We sample from the combined pool of model predictions proportional to each model’s evidence under the assumption that the model priors are equal. To calculate this, we use our Bayes factor estimates and the following formula:

$$\begin{array}{c} \tilde{p}\left(M_{R_{0}}|D\right)=\frac{B_{R_{0},\lambda }}{1+B_{R_{0},\lambda }}, \end{array}$$ (8)

where $\tilde{p}\left(M_{R_{0}}|D\right)$ is the *R*_0_ model evidence under the assumption of equal model priors. We also sample the model predictions proportional to the model evidence where the model priors are as we have defined them; the results of which can be found in the supplementary material.

## Results

We shall first detail the outcome of the estimation for each model and then combine the full predicted distributions to calculate the transmission intensity across the African endemic region and thus the disease burden.

### Estimation of the GLM for YF occurrence

**Fig 4:**
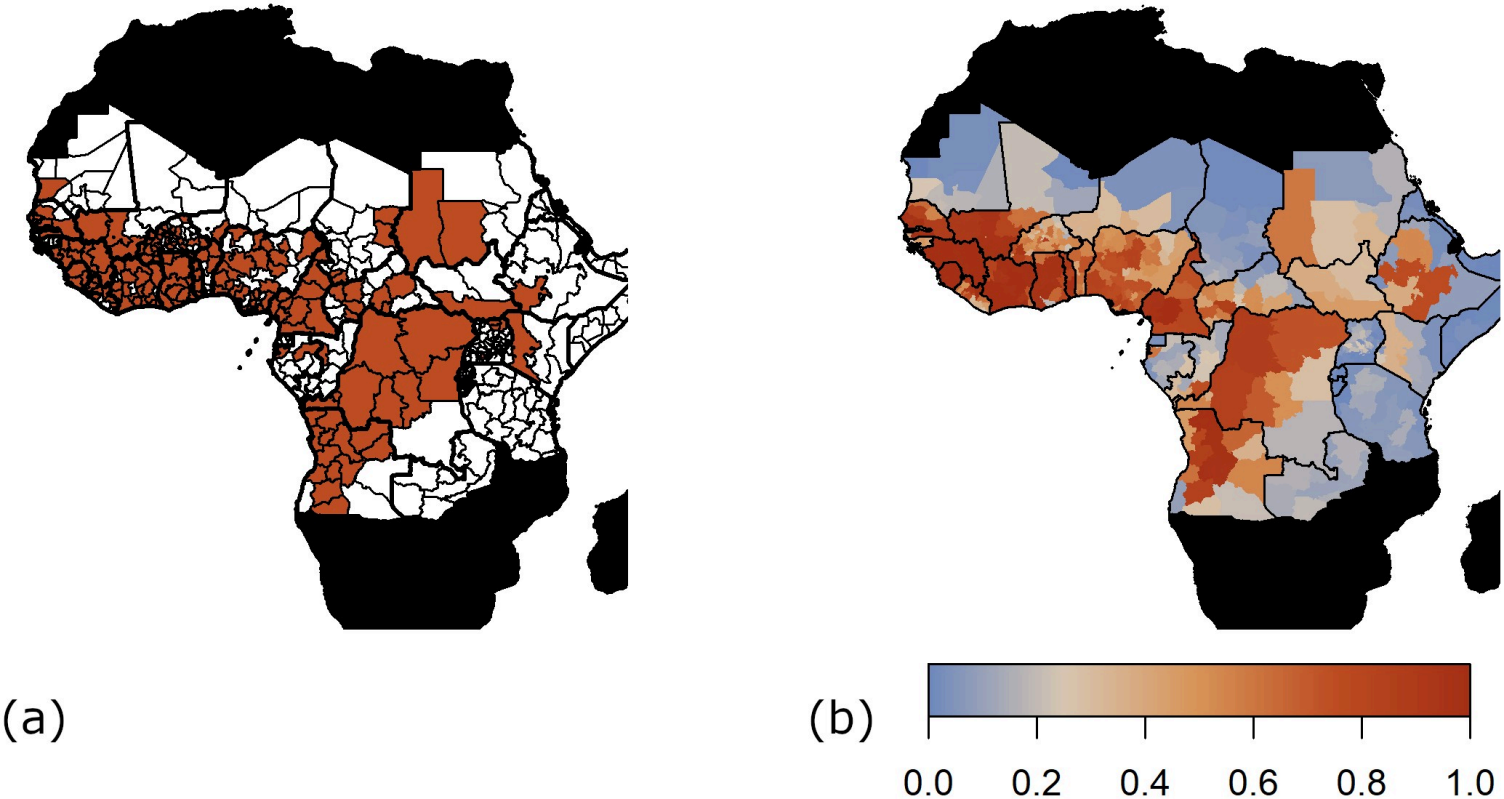
Geographical distribution of yellow fever occurrence. Presence/ absence of yellow fever over 30 year period by province where white indicates absence and brown, presence (a). Median model predictions of the probability of at least one report of yellow fever (b). Countries not considered endemic for yellow fever are shown in black. The AUC of the shown fit is 0.9157. Maps were produced from GADM version 2.0.

The generalised linear model provides a close quantitative and qualitative fit to the data as shown by Fig 4. Stability of the chains was assessed both visually through examination of the trace plots and comparison of the whole and partial chain densities, see S1 Text for figures. Additionally, the Raftery diagnostic was used to assess the number of iterations required to accurately estimate the quantiles of the parameters [36, 37]. All estimation and analyses were performed in R version 3.4.4 [38].

### Estimation of the transmission models

**Fig 5:**
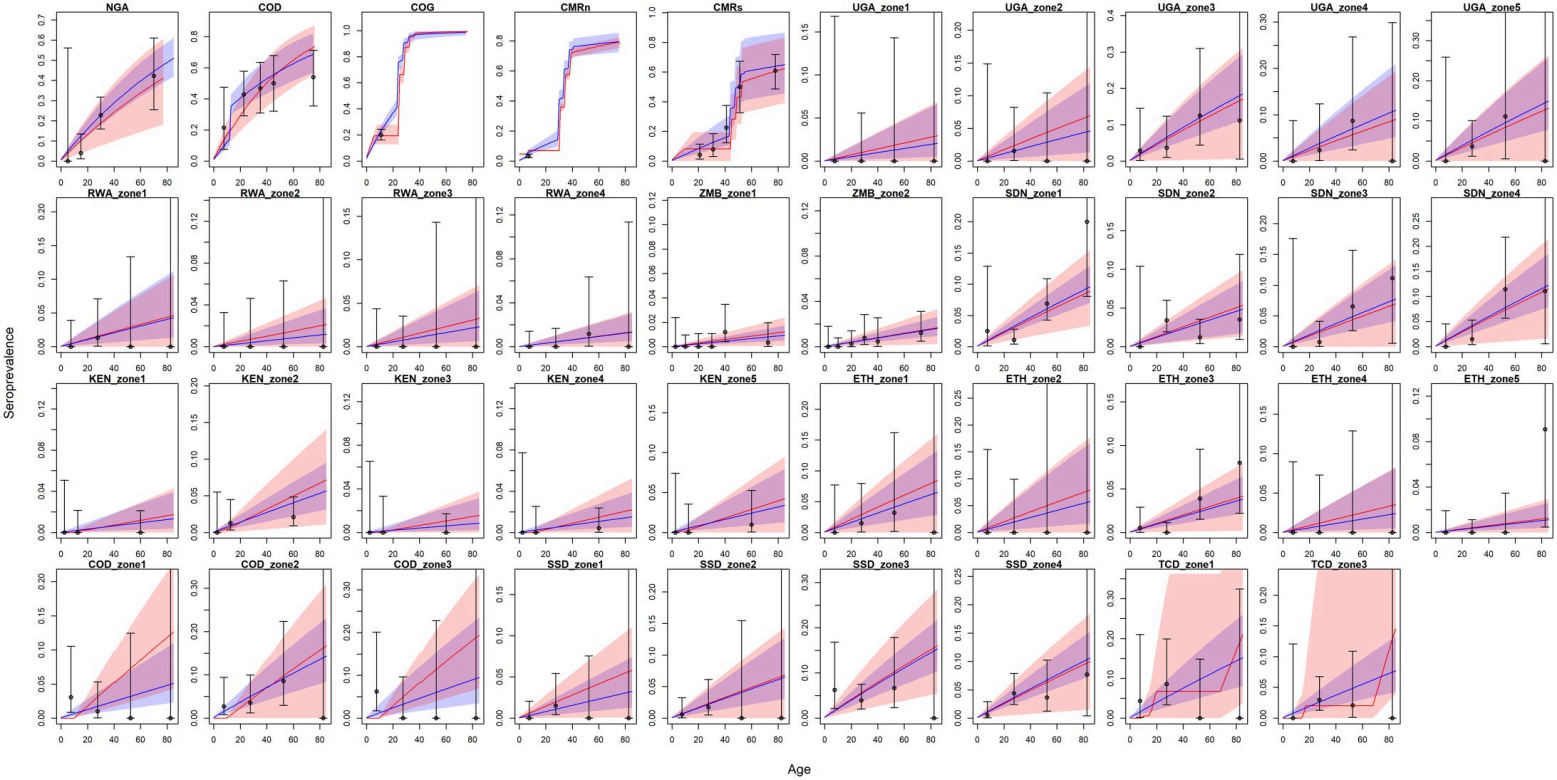
Posterior predictive distributions of seroprevalence for each of the included serological studies. Predictions from the λ model are shown in blue and from the *R*_0_ model, red; paler red and blue regions indicate the 95% credible interval of the predictions. The data is shown with black dots with binomial 95% confidence ranges shown with black whiskers.

The transmission models were estimated together through the product space MCMC method, as such we arrive at marginal posterior distributions for all parameters. In the case of the specific transmission parameters, λ and *R*_0_, these posteriors are independent and so we separate the samples by which model was activated. However, the parameters for vaccine efficacy and vaccine coverage factor for south Cameroon are shared between the models; as such, we include all samples for these parameters to account for uncertainty in model structure in these estimates. The posterior distributions for these shared parameters are shown in Fig C in S1 Text and all other trace plots and distributions can be found in the appendix. Fig 5 shows the posterior predictive distributions for all serological surveys for both transmission models. In the majority of situations, both the λ and *R*_0_ models give a similar qualitative fit to the data. However, there are notable differences in COD zones 1 to 3 where the *R*_0_ model fails to predict the first data point.

### Bayes factors

The Bayes factors are approximated using Eq 7. We defined the prior probability of the λ model as exp(−10) in our specific formulation of the inference, therefore the only additional consideration is the posterior model probability of each model. The approximate posterior probability of the *R*_0_ model is approximately 0.0675 [0.0691, 0.0706] where the confidence interval is provided from a 1,000 bootstrap samples of the model indicator chain [32]. This leads to a log Bayes factor of -12.60 [-12.63,-12.58] for the *R*_0_ model indicating strong evidence for the static transmission force of infection model.

### Bayesian model average predictions

**Fig 6:**
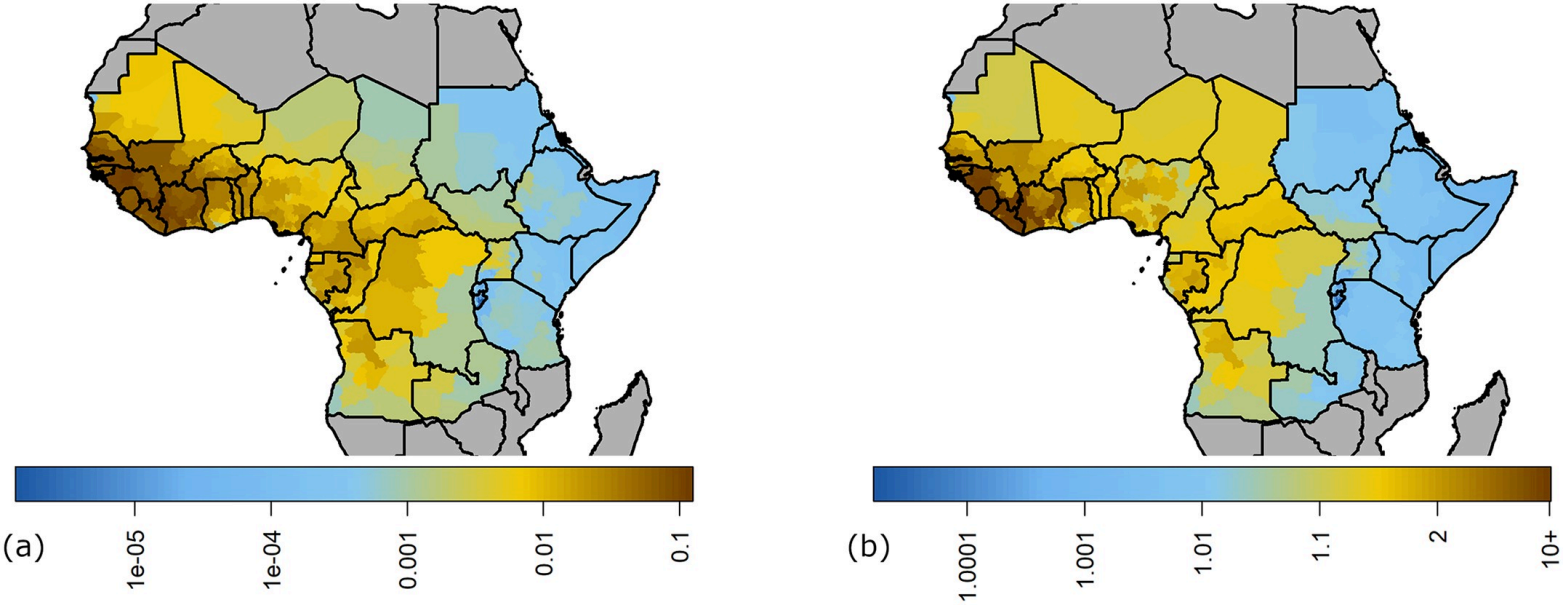
Estimated transmission intensity across the African endemic region for yellow fever. Median posterior estimates of the GLM and transmission model parameters are used to calculate either (a) force of infection or (b) *R*_0_ across the African endemic region. Countries not considered endemic for yellow fever are shown in grey. Maps were produced from GADM version 2.0.

We sample from the posterior distributions of all models to produce estimates of the transmission intensity across the African endemic region, with median values shown in Fig 6. We take 1,000 samples of the posterior for the GLM parameters and use these with 1,000 samples of the transmission models.

**Fig 7:**
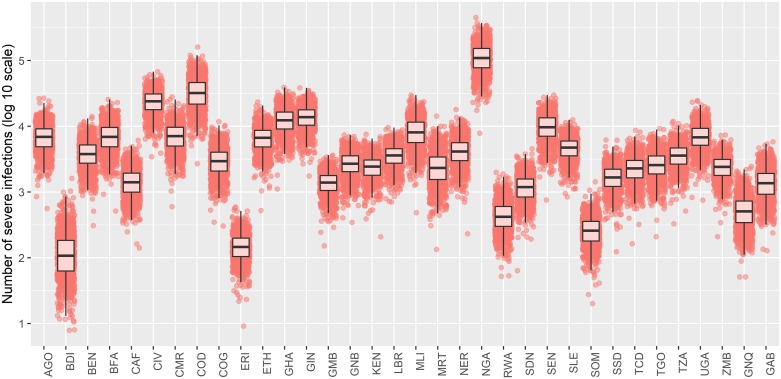
Disease burden estimates for 2018 with equal model priors. 1,000 predictions of the burden in 2018 across the African endemic zone on log10 scale. The probability an infection is severe is drawn from a beta distribution with shape parameters 6.4 and 44.6 [39]. Predictions are drawn from each transmission model proportional to the model evidence under the assumption of equal model priors where pink points come from the λ model and blue points, from the *R*_0_ model.

The transmission models are then sampled proportional to their model evidence under the assumption of equal priors, see Eq 8. In this case, the *R*_0_ model is sampled with probability 3.37×10^−6^, so essentially all 1,000 predictions shown in Fig 7 are obtained from the λ model. We also consider the case where the model priors are as we defined, *π*_λ_ = exp(−10), the resulting burden predictions are shown in Fig K in S1 Text.

## Discussion

We have presented a framework to assess model evidence for two transmission models for yellow fever in Africa. We have used these two models in conjunction with a model of relative risk of yellow fever reports to estimate transmission intensity across the African endemic region. We were then able to produce average model predictions of the burden across the region according to each model’s evidence.

We found strong support for the static force of infection model with a Bayes factor of 2.95 ×10^5^ (95% CrI [2.95 ×10^5^, 2.95 ×10^5^]). This suggests that for the currently included serological surveys, there is more support for the static force of infection model. This could be due to unsuitability of the *R*_0_ model for the type of data we have access to or the sparsity of the data itself; however, it could also indicate the relative validity of assumptions concerning yellow fever transmission in Africa.

Whilst the models we compare have some similar structure, the intrinsic assumptions in each differ. We have omitted one serological survey, from the Central African Republic, that was included in the original estimation of both transmission models as it may not represent the population level immunity at steady state [1, 9, 18]. When this survey was included, the support for the *R*_0_ model decreased, suggesting that the assumption of constant transmission intensity disproportionally hampers the *R*_0_ model. The *R*_0_ model also assumes that each province is at endemic equilibrium. This assumption may be too strong in East Africa where very low values of *R*_0_ are estimated. It also leads to some years where there are no new infections in certain provinces, see COD zones 1 to 3 in Fig 5 and in the burden predictions in Fig K in S1 Text. Furthermore, changes to the demography could affect the endemic equilibrium making the *R*_0_ less flexible than the static force of infection model. Finally, by fitting the same model indicator over all locations, we implicitly assume that the relative support for each model is spatially invariant. As such, this could penalise areas where the *R*_0_ is better supported.

The example of COD zones 1-3 suggests that the disparity between models is being driven by specific locations. In these situations, the population level immunity is close to herd immunity, leading to years of zero infections in the *R*_0_ model when vaccine coverages are higher; a behaviour never seen in the λ model. As both models are designed to assess long-term disease burden, this behaviour is smoothed over the timespan; however, when comparing specific age groups in serological profiles, the differences can be seen. This behaviour leads to large differences in pseudo- log- likelihood for some age groups in the serology, leading to the large magnitude of the Bayes factor. It also means that the Bayes factor is dependant on the included studies.

If our model assumptions were directly comparable, then we may conclude that the λ model provides a stronger representation of yellow fever transmission in Africa. This would suggest transmission occurring as a result of a constant pressure from the sylvatic reservoir may be responsible for more occurrence reports than human to human transmission (mediated by mosquitoes). This would be consistent with the dynamics seen for the ongoing Nigerian yellow fever outbreak, but not for the 2016 Angola outbreak [9]. However, the way in which we incorporate the available data into our model may contribute to the relative support.

Untangling the relative contribution of transmission routes may not be feasible with our current data sets. Our results are limited by the number of compared transmission models and the sparse data set on which to compare them. We could counter the first issue by introducing further transmission models that bridge the two approaches. However, the second issue will limit how any new models may be estimated. We have utilised occurrence data and serological studies. To show occurrence, we take cases and outbreak reports only, discarding the relative magnitude of outbreaks, as we expect any reports to be a large underestimate of the actual prevalence of the disease. This may indirectly favour the static force of infection model as infections are assumed to be unconnected. In the serological studies, there are key gaps, particularly in West Africa where transmission is estimated to be highest. The absence of information is this region both increases the uncertainty in our estimates and may mean we omit areas where urban transmission plays a greater role. One of the benefits of the framework we use is accounting for some of the uncertainty arising from these data gaps. Yet, we can not ensure that both models will be affected equally.

Our framework for model comparison and averaging may be extended to other models given they share data sources. We have presented a method for dealing with pseudo-likelihoods within a product space estimation format. This leads to posterior estimates of model parameters and model evidence whilst accounting for uncertainty in model structure. We find strong evidence for a model with constant infection pressure from an external source, validating proposals that yellow fever is sustained in a sylvatic reservoir. However these results are limited by the small number of models compared and the sparse data available. New studies and data collection, particularly serological data in West Africa, could reduce uncertainty and alter the current model evidence. As an increasing number of models are developed for disease transmission, rigorous comparison accounting for uncertainty in parameter estimates and model structure is vital in order for us to unpack the relative importance of transmission dynamics. We present one avenue of model comparison and highlight areas of uncertainty both in yellow fever modelling and available yellow fever data.

## Supporting information

S1 Text Supporting text. Additional data and estimation figures as well as further explanation of the main text.
(PDF)

## References

[1] Garske T, Van Kerkhove MD, Yactayo S, Ronveaux O, Lewis RF, Staples JE, et al Yellow fever in Africa: estimating the burden of disease and impact of mass vaccination from outbreak and serological data. PLoS Medicine. 2014;11(5):e1001638 10.1371/journal.pmed.1001638 24800812 PMC4011853

[2] LaBeaud A, Bashir F, King CH. Measuring the burden of arboviral diseases: the spectrum of morbidity and mortality from four prevalent infections. Population health metrics. 2011;9(1):1 10.1186/1478-7954-9-1 21219615 PMC3024945

[3] Monath TP, Vasconcelos PF. Yellow fever. Journal of Clinical Virology. 2015;64:160–173. 10.1016/j.jcv.2014.08.030 25453327

[4] Barrett AD, Higgs S. Yellow fever: a disease that has yet to be conquered. Annu Rev Entomol. 2007;52:209–229. 10.1146/annurev.ento.52.110405.091454 16913829

[5] Gotuzzo E, Yactayo S, Córdova E. Efficacy and duration of immunity after yellow fever vaccination: systematic review on the need for a booster every 10 years. The American Journal of Tropical Medicine and Hygiene. 2013;89(3):434–444. 10.4269/ajtmh.13-0264 24006295 PMC3771278

[6] Monath TP, Nasidi A. Should yellow fever vaccine be included in the expanded program of immunization in Africa? A cost-effectiveness analysis for Nigeria. The American journal of tropical medicine and hygiene. 1993;48(2):274–299. 10.4269/ajtmh.1993.48.274 8447531

[7] Shearer FM, Longbottom J, Browne AJ, Pigott DM, Brady OJ, Kraemer MU, et al Existing and potential infection risk zones of yellow fever worldwide: a modelling analysis. The Lancet Global Health. 2018 10.1016/S2214-109X(18)30024-X 29398634 PMC5809716

[8] Shearer FM, Moyes CL, Pigott DM, Brady OJ, Marinho F, Deshpande A, et al Global yellow fever vaccination coverage from 1970 to 2016: an adjusted retrospective analysis. The Lancet infectious diseases. 2017;17(11):1209–1217. 10.1016/S1473-3099(17)30419-X 28822780 PMC5666204

[9] Jean K, Hamlet A, Benzler J, Cibrelus L, Gaythorpe KAM, Sall A, et al. Eliminating yellow fever epidemics in Africa: vaccine demand forecast and impact modelling. bioRxiv. 2018. 10.1371/journal.pntd.0008304 PMC7237041 32379756

[10] Faria NR, Kraemer MU, Hill S, De Jesus JG, Aguiar R, Iani FC, et al Genomic and epidemiological monitoring of yellow fever virus transmission potential. Science. 2018;361(6405):894–899. 10.1126/science.aat7115 30139911 PMC6874500

[11] Hamlet A, Jean K, Yactayo S, Benzler J, Cibrelus L, Ferguson N, et al POLICI: A web application for visualising and extracting yellow fever vaccination coverage in Africa. Vaccine. 2019;37(11):1384–1388. 10.1016/j.vaccine.2019.01.074 30770224

[12] Moreau J, Girault G, Drame I, Perraut R. Reemergence of yellow fever in West Africa: lessons from the past, advocacy for a control program. Bulletin de la Societe de pathologie exotique (1990). 1999;92(5):333–336. 10690471

[13] Durieux C. Mass yellow fever vaccination in French Africa south of the Sahara. Yellow Fever Vaccination, Monograph Series. 1956;30:115–121.

[14] World Health Organization. The weekly epidemiological record (WER).

[15] World Health Organization. Disease outbreak news (DON).

[16] World Health Organization. The yellow fever initiative: an introduction. 2016.

[17] World Health Organization/ UNICEF. Estimates of National Immunization Coverage (WUENIC) 2015;. Available from: http://apps.who.int/immunization_monitoring/globalsummary/timeseries/tswucoverageyfv.html.

[18] Rapid assessment of Yellow Fever viral activity in the Central African Republic.

[19] Kuniholm MH, Wolfe ND, Huang CYh, Mpoudi-Ngole E, Tamoufe U, Burke DS, et al Seroprevalence and distribution of Flaviviridae, Togaviridae, and Bunyaviridae arboviral infections in rural Cameroonian adults. The American Journal of Tropical Medicine and Hygiene. 2006;74(6):1078–1083. 10.4269/ajtmh.2006.74.1078 16760524

[20] Merlin M, Josse R, Kouka-Bemba D, Meunier D, Senga J, Simonkovich E, et al Evaluation of immunological and entomotological indices of yellow fever in Pointe-Noire, People’s Republic of Congo. Bulletin de la Societe de pathologie exotique et de ses filiales. 1986;79(2):199–206. 3731366

[21] Omilabu S, Adejumo J, Olaleye O, Fagbami A, Baba S. Yellow fever haemagglutination-inhibiting, neutralising and IgM antibodies in vaccinated and unvaccinated residents of Ibadan, Nigeria. Comparative Immunology, Microbiology and Infectious Diseases. 1990;13(2):95–100. 10.1016/0147-9571(90)90521-T 2208973

[22] Tsai T, Lazuick JS, Ngah R, Mafiamba P, Quincke G, Monath TP. Investigation of a possible yellow fever epidemic and serosurvey for flavivirus infections in northern Cameroon, 1984. Bulletin of the World Health Organization. 1987;65(6):855 3501739 PMC2491080

[23] Werner G, Huber H, Fresenius K. Prevalence of yellow fever antibodies in north Zaire. In: Annales de la Societe belge de medecine tropicale. vol. 65; 1984 p. 91–93. 4004378

[24] Tsegaye MM, Beyene B, Ayele W, Abebe A, Tareke I, Sall A, et al Sero-prevalence of yellow fever and related Flavi viruses in Ethiopia: a public health perspective. BMC public health. 2018;18(1):1011 10.1186/s12889-018-5726-9 30107830 PMC6092792

[25] Garske T, Ferguson NM, Ghani AC. Estimating air temperature and its influence on malaria transmission across Africa. PLoS One. 2013;8(2):e56487 10.1371/journal.pone.0056487 23437143 PMC3577915

[26] United Nations DoE. Projections S. World Population Prospects.

[27] Dobson JE, Bright EA, Coleman PR, Durfee RC, Worley BA. LandScan: a global population database for estimating populations at risk. Photogrammetric engineering and remote sensing. 2000;66(7):849–857.

[28] NASA LD. NASA Land Processes Distributed Active Archive Center (LP DAAC) USGS/Earth Resources Observation and Science (EROS) Center; 2001.

[29] Xie P, Arkin PA. Analyses of global monthly precipitation using gauge observations, satellite estimates, and numerical model predictions. Journal of climate. 1996;9(4):840–858. 10.1175/1520-0442(1996)009<0840:AOGMPU>2.0.CO;2

[30] Hijmans R, Cameron S, Parra J, Jones P, Jarvis A. The WorldClim interpolated global terrestrial climate surfaces. Version 1.3; 2004.

[31] Carlin BP, Chib S. Bayesian model choice via Markov chain Monte Carlo methods. Journal of the Royal Statistical Society Series B (Methodological). 1995; p. 473–484. 10.1111/j.2517-6161.1995.tb02042.x

[32] Lodewyckx T, Kim W, Lee MD, Tuerlinckx F, Kuppens P, Wagenmakers EJ. A tutorial on Bayes factor estimation with the product space method. Journal of Mathematical Psychology. 2011;55(5):331–347. 10.1016/j.jmp.2011.06.001

[33] Green P, O’Hagan A. Model choice with MCMC on product spaces without using pseudo-priors. Department of Mathematics, University of Nottingham. 1998.

[34] Godsill SJ. On the relationship between Markov chain Monte Carlo methods for model uncertainty. Journal of Computational and Graphical Statistics. 2001;10(2):230–248. 10.1198/10618600152627924

[35] Dellaportas P, Forster JJ, Ntzoufras I. On Bayesian model and variable selection using MCMC. Statistics and Computing. 2002;12(1):27–36. 10.1023/A:1013164120801

[36] Raferty A, Lewis S. The number of iterations, convergence diagnostics and generic Metropolis algorithms. Practical Markov Chain Monte Carlo. 1995.

[37] Raffery A, Lewis S. One long run with diagnostics: Implementation strategies for Markov chain Monte Carlo. Statist Sci. 1992;7:493–497. 10.1214/ss/1177011143

[38] R Core Team. R: A Language and Environment for Statistical Computing; 2013 Available from: http://www.R-project.org/.

[39] Johansson MA, Vasconcelos PF, Staples JE. The whole iceberg: estimating the incidence of yellow fever virus infection from the number of severe cases. Transactions of The Royal Society of Tropical Medicine and Hygiene. 2014;108(8):482–487. 10.1093/trstmh/tru092 24980556 PMC4632853

