# Supplementary material for: Quantifying model evidence for yellow fever transmission routes in Africa
Source: PLoS Comput Biol. 2019 Sep 23;15(9):e1007355. doi: 10.1371/journal.pcbi.1007355 (PMC6779277; doi:10.1371/journal.pcbi.1007355)
Supplement: S1 Text — Additional data and estimation figures as well as further explanation of the main text. (PDF) [file pcbi.1007355.s001.pdf]

Quantifying model evidence for yellow fever transmission routes in Africa: Supporting Information

K. A. M. Gaythorpe [1*], K. Jean[2], L. Cibrelus [3], T. Garske[1]

1 School of Public Health/ Department of Medicine/ Imperial College London/ UK
2 Laboratoire MESuRS/ CNAM Paris/ France
3 Infectious Hazard Management department/ World Health Organisation/ Geneva/ Switzerland

* k.gaythorpe@imperial.ac.uk

1 Data

1.1 Serological surveys

Survey location	Sample size	Year	Age range	Reference
Nigeria	184	1990	0-100	[1]
Central African Republic	938	2009	0-100	[2]
Democratic republic of Congo	140	1985	0-100	[3]
Republic of Congo	360	1985	0-100	[4]
Cameroon (North)	1987	840	0-14	[5]
Cameroon (South)	2001	256	0-100	[6]
Uganda (zones)	584	2012	0-100	
Rwanda (zones)	1286	2012	0-100	
Zambia (zones)	3679	2013	0-100	
Sudan (zones)	1814	2012	0-100	
Kenya (zones)	1960	2013	0-100	
Ethiopia (zones)	1645	2014	0-100	[7]
Democratic republic of Congo (zones)	479	2014	0-100	
South Sudan (zones)	1480	2014	0-100	
Chad (zones)	352	2014	0-100	

Table A. Characteristics of included serological surveys.

2 Estimation

For the main estimation included in the manuscript, 6 MCMC chains were run for 200,000 iterations each. These were a result of multiple estimation runs including testing with different serological surveys included and with different start conditions or model prior. For the GLM, one chain was run for 400,000 iterations and compared to estimation for convergence. This was separate from the product space estimation method.

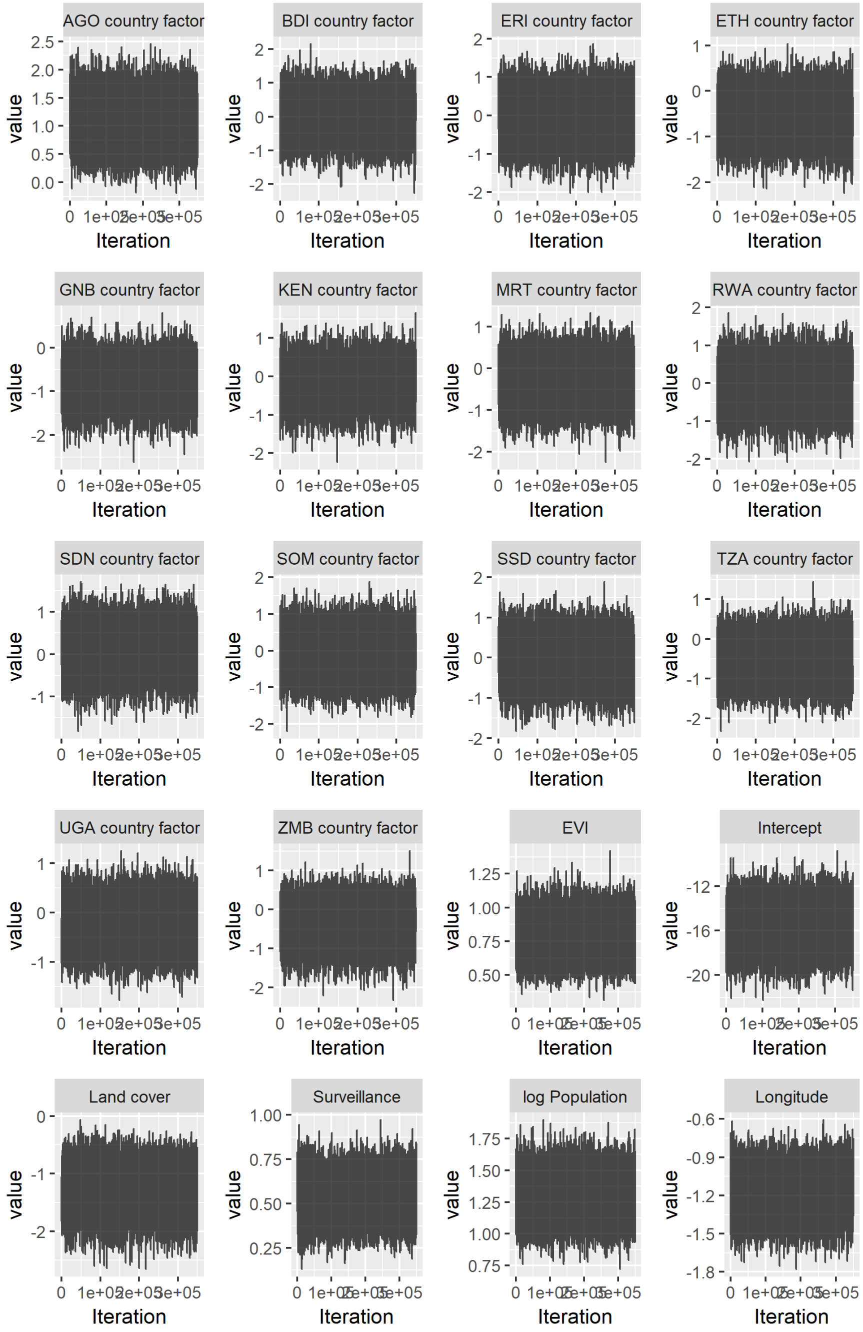

Fig A. Trace plots of the estimated GLM parameters. Plotted with use of ggmcmc package [8].

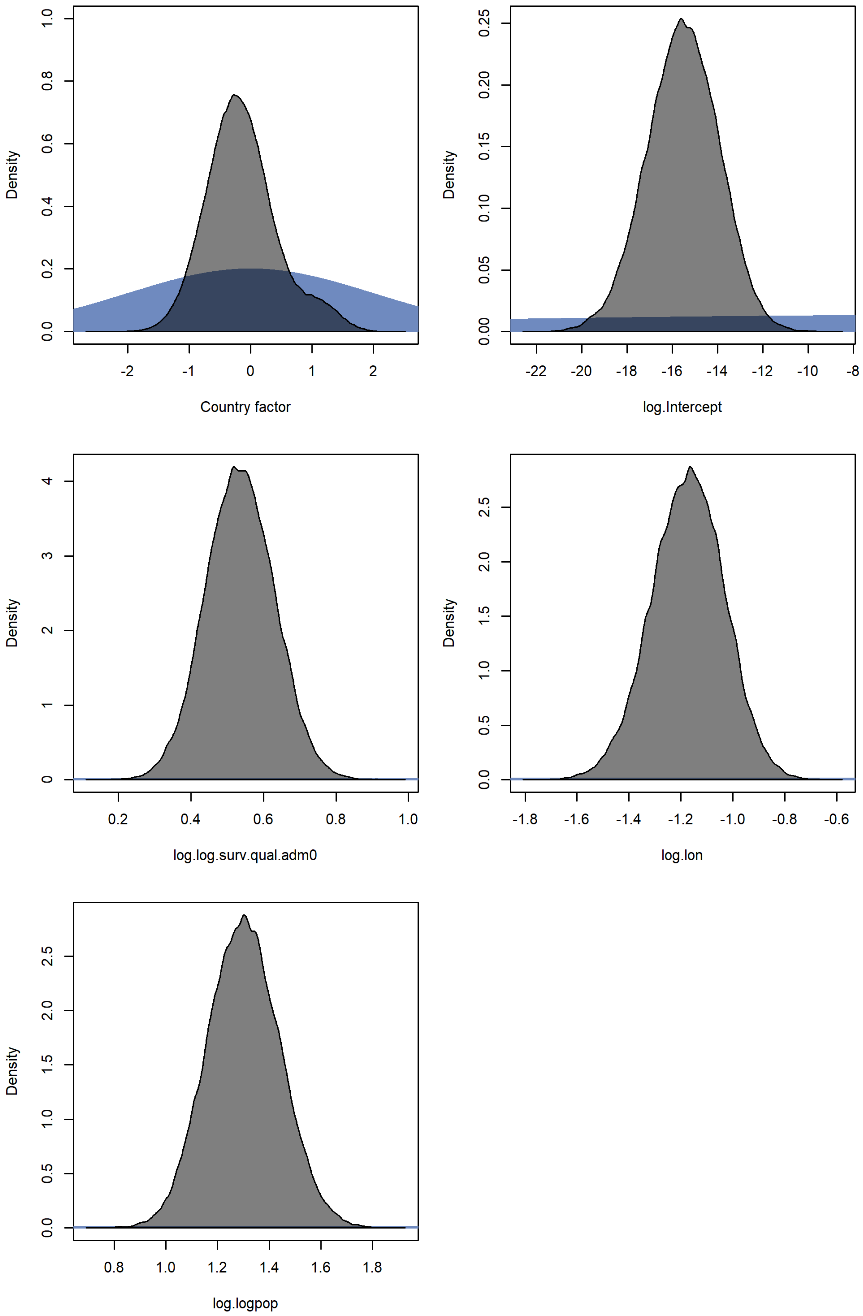

Fig B. Prior and posterior of GLM parameters. Prior (blue) and posterior (grey) distributions for all parameters in the GLM.

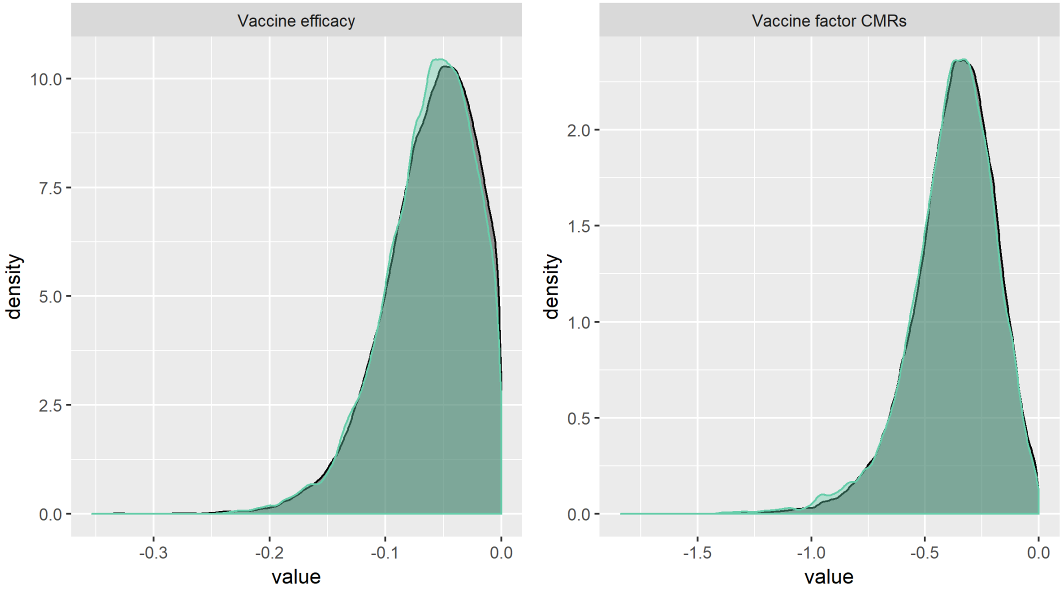

Fig C. Comparison of MCMC chain for shared parameters in transmission models. Whole chain (black) and last 10% of chain (green) distributions for shared parameters in the transmission models.

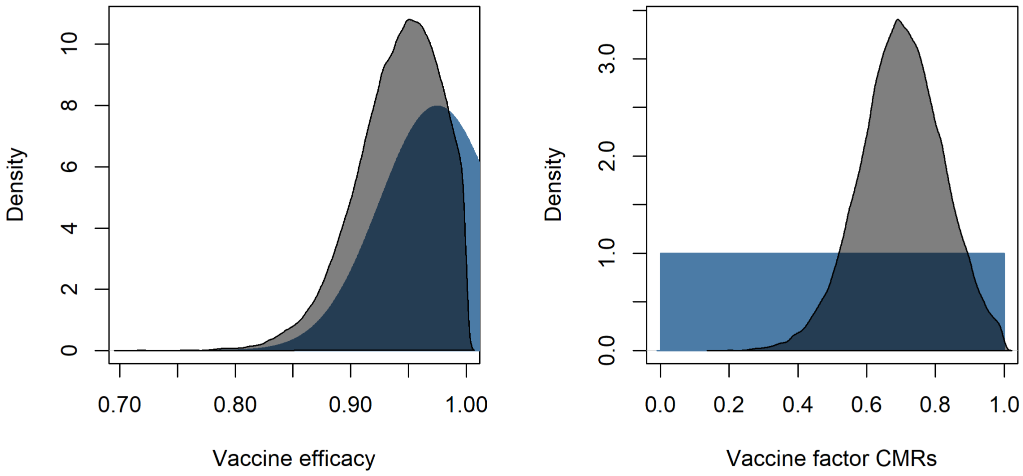

Fig D. Prior (blue) and posterior (black) distributions for shared parameters in transmission model.

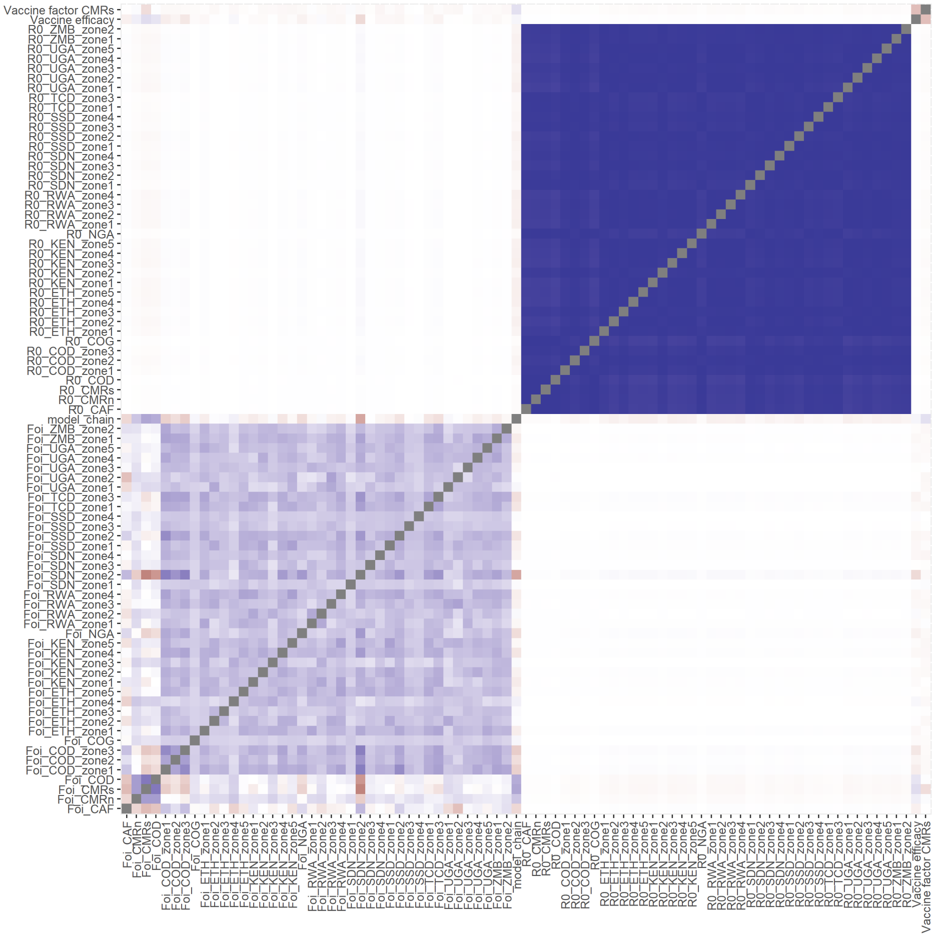

Fig E. Cross correlation plot where red indicates positive, and blue, negative, correlations.

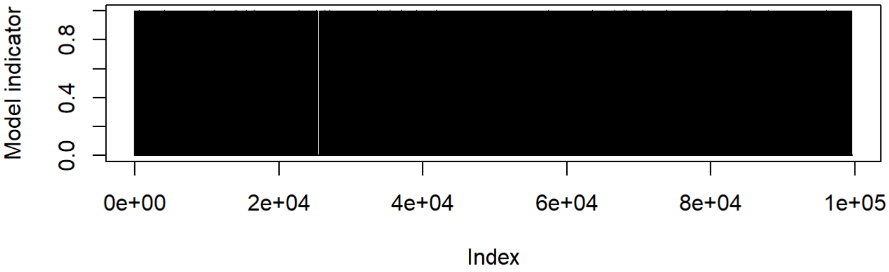

Fig F. MCMC chain for the model indicator.

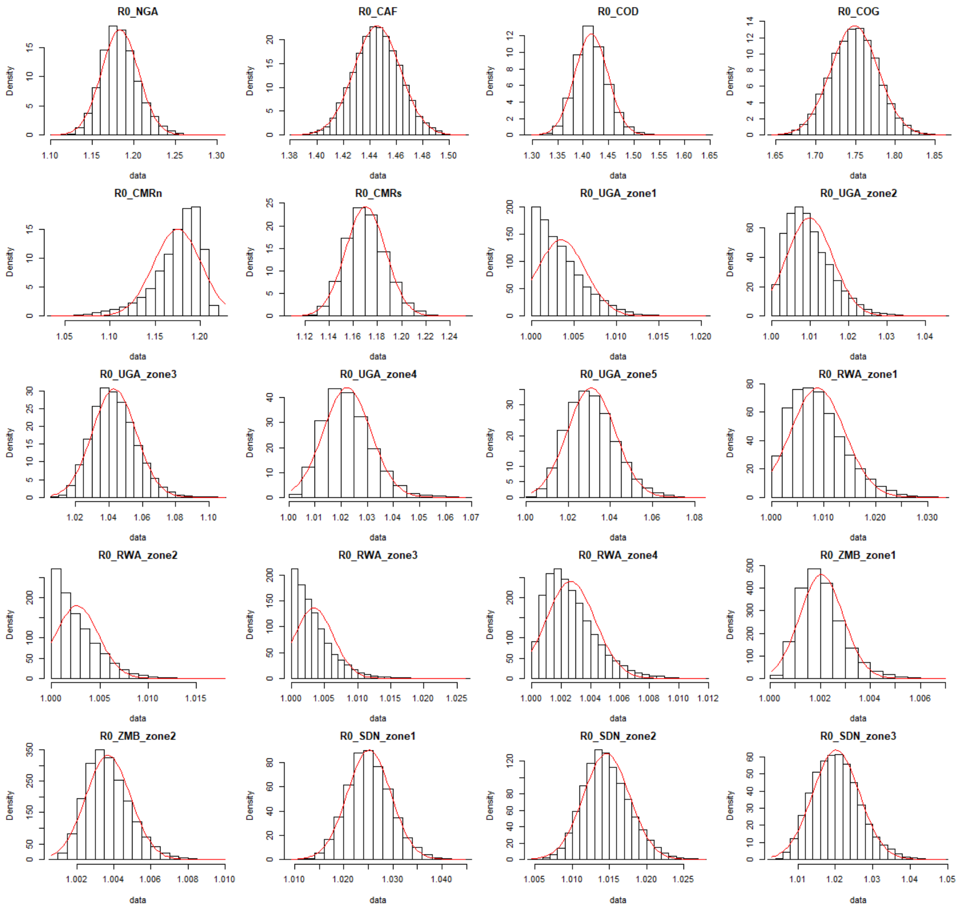

Fig G. Pseudoprior fit R_0 surveys 1:20.

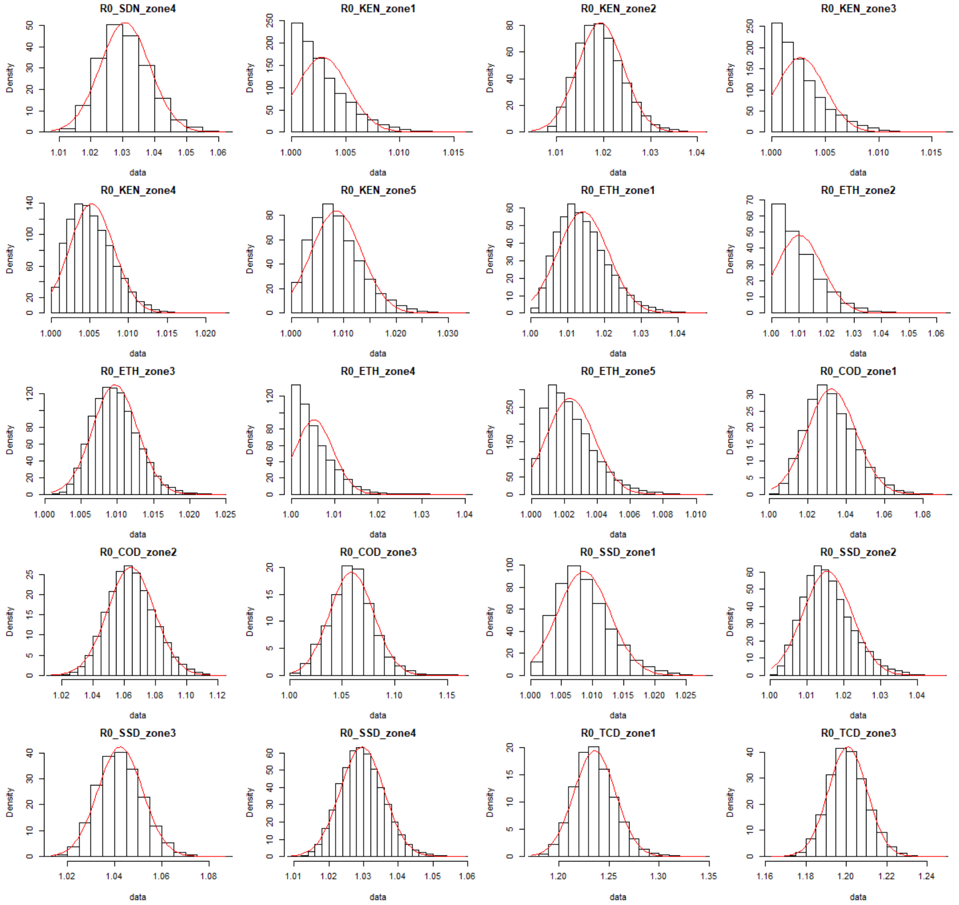

Fig H. Pseudoprior fit R_0 surveys 21:40.

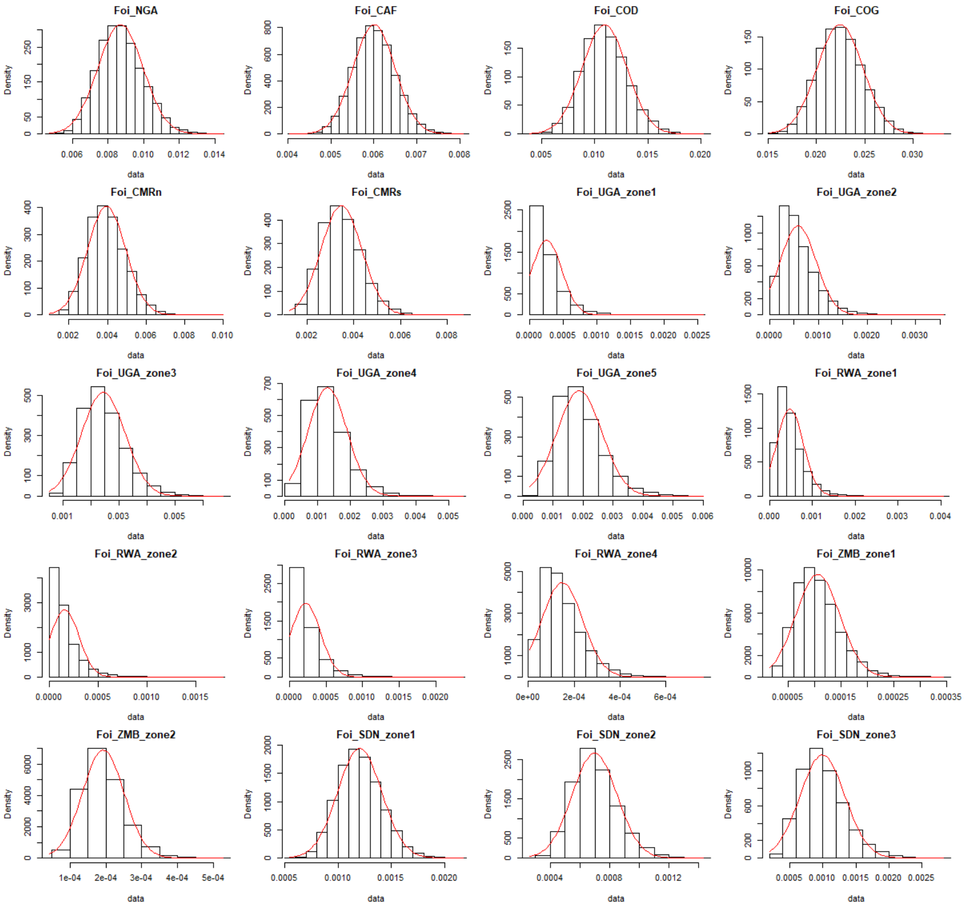

Fig I. Pseudoprior fit λ surveys 1:20.

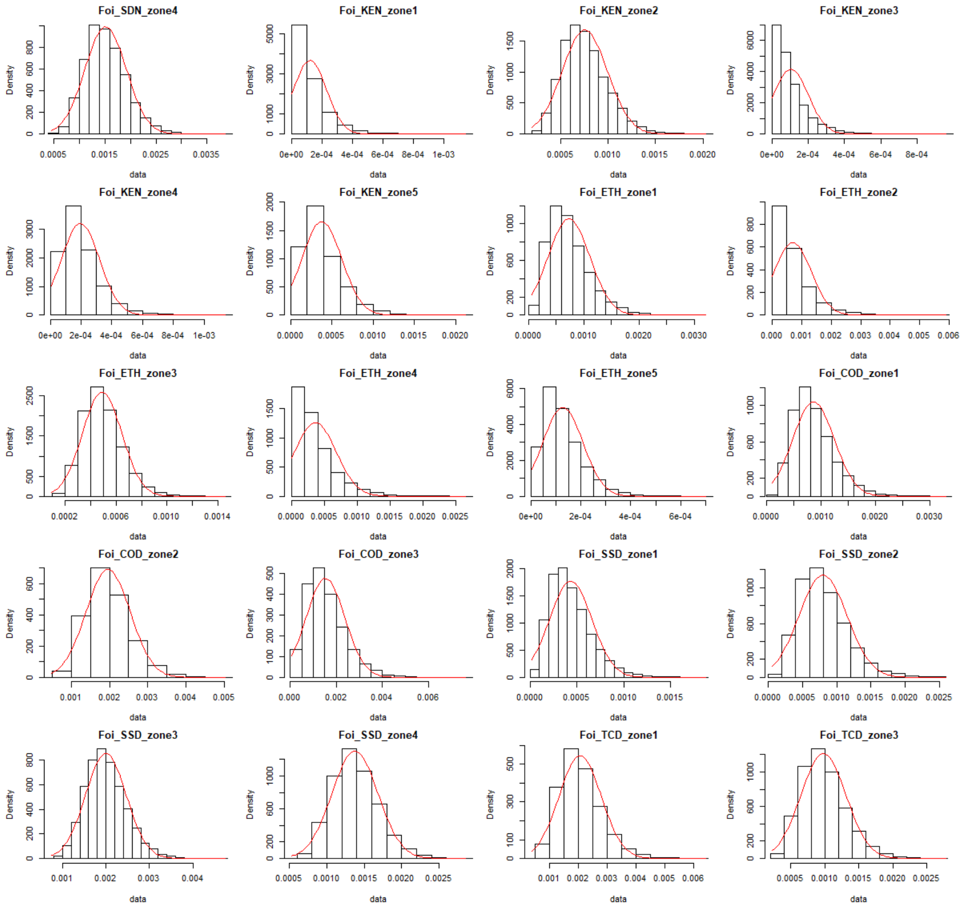

Fig J. Pseudoprior fit λ surveys 21:40.

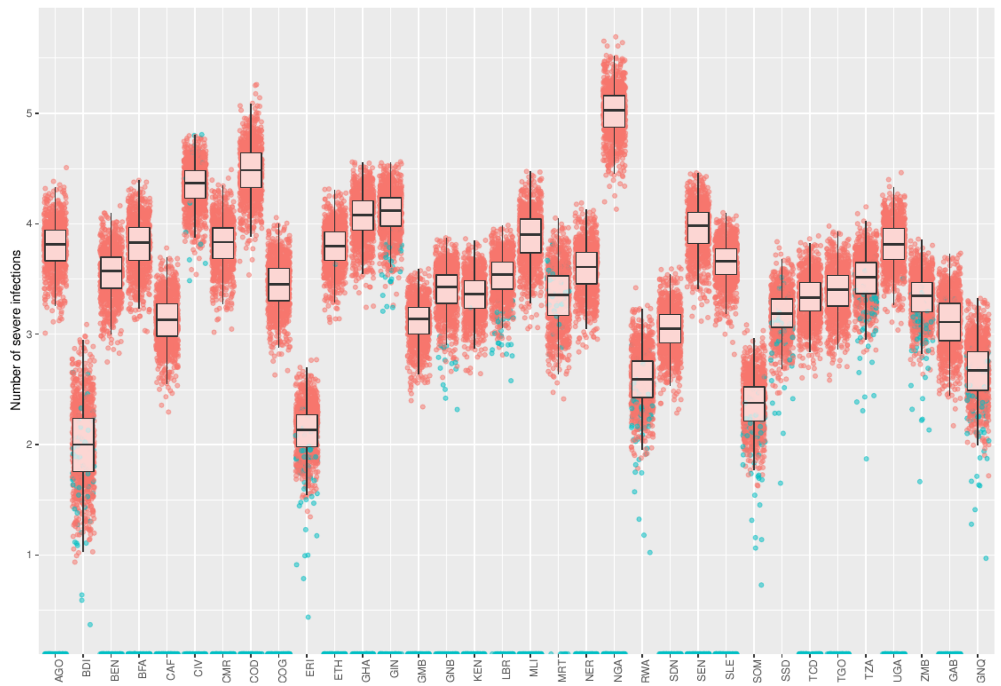

Fig K. Log disease burden estimates for 2018. 1,000 predictions of the burden in 2018 across the African endemic zone on log10 scale. The probability that an infection is severe is drawn from a beta distribution with shape parameters 6.4 and 44.6 [9]. Predictions are drawn from each transmission model proportional to the model evidence as estimated from our posterior model probability where pink points come from the λ model and blue points, from the R_0 model.

References

1. Omilabu S, Adejumo J, Olaleye O, Fagbami A, Baba S. Yellow fever haemagglutination-inhibiting, neutralising and IgM antibodies in vaccinated and unvaccinated residents of Ibadan, Nigeria. Comparative Immunology, Microbiology and Infectious Diseases. 1990;13(2):95–100.

2. Staples JE, Diallo M, Janusz KB, Manengu C, Lewis RF, Perea W, et al. Yellow fever risk assessment in the Central African Republic. Transactions of the Royal Society of Tropical Medicine and Hygiene. 2014;108(10):608–615.

3. Werner G, Huber H, Fresenius K. Prevalence of yellow fever antibodies in north Zaire. In: Annales de la Societe belge de medecine tropicale. vol. 65; 1984. p. 91–93.

4. Merlin M, Josse R, Kouka-Bemba D, Meunier D, Senga J, Simonkovich E, et al. Evaluation of immunological and entomotological indices of yellow fever in Pointe-Noire, People's Republic of Congo. Bulletin de la Societe de pathologie exotique et de ses filiales. 1986;79(2):199–206.

5. Tsai T, Lazuick JS, Ngah R, Mafiamba P, Quincke G, Monath TP. Investigation of a possible yellow fever epidemic and serosurvey for flavivirus infections in northern Cameroon, 1984. Bulletin of the World Health Organization. 1987;65(6):855.

6. Kuniholm MH, Wolfe ND, Huang CYh, Mpoudi-Ngole E, Tamoufe U, Burke DS, et al. Seroprevalence and distribution of Flaviviridae, Togaviridae, and Bunyaviridae arboviral infections in rural Cameroonian adults. The American Journal of Tropical Medicine and Hygiene. 2006;74(6):1078–1083.

7. Tsegaye MM, Beyene B, Ayele W, Abebe A, Tareke I, Sall A, et al. Sero-prevalence of yellow fever and related Flavi viruses in Ethiopia: a public health perspective. BMC public health. 2018;18(1):1011.

8. i Marín XF. GGMCMC: Analysis of MCMC Samples and Bayesian Inference. Journal of Statistical Software. 2016;70(9):1–20. doi:10.18637/jss.v070.i09.

9. Johansson MA, Vasconcelos PF, Staples JE. The whole iceberg: estimating the incidence of yellow fever virus infection from the number of severe cases. Transactions of The Royal Society of Tropical Medicine and Hygiene. 2014;108(8):482–487.
